# Pericardial Tamponade in an Adult Suffering from Acute Mumps Infection

**DOI:** 10.1155/2016/7980936

**Published:** 2016-10-12

**Authors:** Sascha Kahlfuss, Robert Rainer Flieger, Annette Mankertz, Kadir Yilmaz, Torsten Kai Roepke

**Affiliations:** ^1^Department of Cardiology, HELIOS Clinical Medical Center Köthen, 06366 Köthen, Germany; ^2^National Reference Center for Measles, Mumps and Rubella, Robert Koch Institute, 13353 Berlin, Germany; ^3^Experimental and Clinical Research Center, Charité-Universitätsmedizin Berlin, 13125 Berlin, Germany

## Abstract

Here, we report a case of a 51-year-old man with acute pericardial tamponade requiring emergency pericardiocentesis after he suffered from sore throat, headache, malaise, and sweats for two weeks. Serological analyses revealed increased mumps IgM and IgG indicating an acute mumps infection whereas other bacterial and viral infections were excluded. In addition, MRI revealed atypical swelling of the left submandibular gland. Whereas mumps has become a rare entity in children due to comprehensive vaccination regimens in western civilizations, our case highlights mumps as an important differential diagnosis also in adults, where the virus can induce life-threatening complications such as pericardial tamponade.

## 1. Introduction

Mumps virus belongs to the family of Paramyxoviridae, genus* Rubulavirus*, and comprises a single-stranded negative-sense RNA of 15,384 nucleotides [1, 2]. As a consequence of comprehensive vaccination strategies in industrialized countries, the incidence of mumps infections is decreasing, although isolated mumps epidemics especially in community facilities are still reported [2, 3]. Importantly, mumps infection provides lifelong immunity, although reinfection by other mumps virus strains or nonresponding to the vaccination appears to be possible [1–4]. Today, more frequently, teenagers and adults (with the highest incidence in 15–25-year-olds) are affected by acute mumps infections [2, 3]. Approximately, one-third of mumps cases take a subclinical course, whereas 70% of the patients develop symptoms such as a respiratory syndrome or unilateral or bilateral (painful) swollen parotid or other salivary glands. Additionally, the disease can evoke complications such as meningitis (10%) or pancreatitis (4%). Other complications include orchitis (15%–30%), oophoritis (5%), or deafness (4%) [1, 5]. Less often mumps virus induces pneumonia and myocarditis [6, 7]. In line with this, we are only aware of a few cases reporting cardiac involvement during mumps infection, although (peri)myocarditis, arrhythmias, or acute and chronic heart failure can be life limiting and, thereby, display fatal complications of an acute mumps infection [6–10].

## 2. Case Report

In 2016, a 51-year-old man presented to our emergency department with dyspnea (New York Heart Association IV) and chest pain (Canadian Cardiovascular Society III) for 2 days. Two weeks earlier, he suffered from progressive fatigue, headache, sore throat, malaise, weight loss, and occasionally sweats. Medical history involved essential arterial hypertension and a mild traumatic brain injury and lung contusion in 2010 after falling from a ladder.

**Figure 1:**
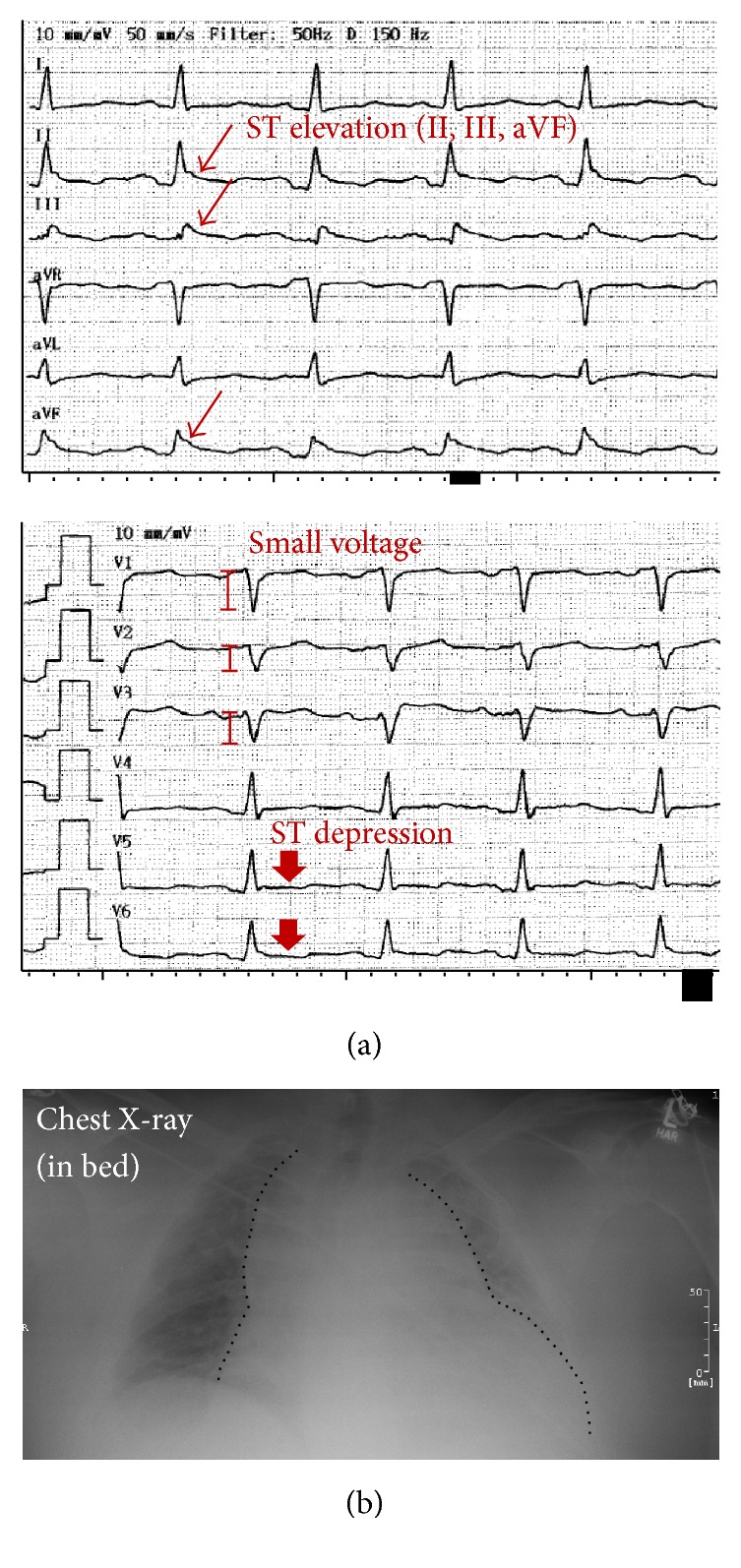
ECG and chest X-ray of the patient. (a) ECG showing ST elevations and low voltage QRS complexes as indirect signs of acute pericardial effusion. (b) Chest X-ray shows cardiomegaly with central and peripheral pulmonary venous congestion. Heart silhouette is highlighted by a dotted black line.

**Figure 2:**
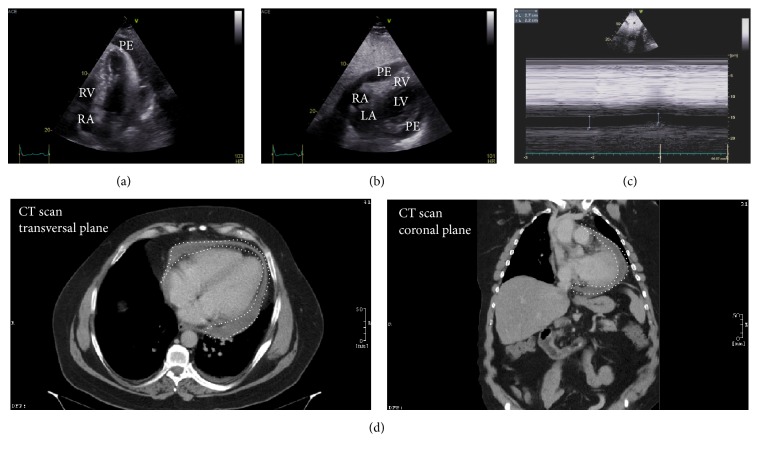
TTE demonstrating intermediate pericardial effusion leading to pericardial tamponade. (a) Apical four-chamber view showing intrapericardial pressure exceeding RA systolic pressure as evidenced by systolic RA collapse. (b) Subcostal view demonstrating diastolic RV collapse. (c) M-mode echocardiography of dilated inferior vena cava (27 mm) with <50% inspiratory reduction. (d) CT with contrast agent reveals residual circumferential pericardial effusion after pericardiocentesis. Pericardial effusion is highlighted by a dotted white line. Structures: pericardial effusion (PE), right atrium (RA), right ventricle (RV), left atrium (LA), and left ventricle (LV).

Electrocardiography (ECG) showed ST elevations in II, III, and aVF as well as low voltage QRS complexes in V_1_–V_3_ (Figure 1(a)). In addition, the patient exhibited tachycardia (103 bpm) and presented with arterial hypotension (90/60 mmHg). Initial laboratory analyses revealed elevated C-reactive protein (CRP, 135 mg/L, <5 mg/L), leukocytosis (21 G/L, 4.0–9.0 G/L), and slightly elevated high-sensitivity troponin I values (0.05 *μ*g/L, <0.03 *μ*g/L). In addition, procalcitonin (PCT) appeared to be increased (0.13 *μ*g/L, <0.05 *μ*g/L). Chest X-ray showed cardiomegaly with peripheral and central pulmonary venous congestion (Figure 1(b)). Transthoracic echocardiography (TTE) revealed a hypertrophied (interventricular septum end-diastolic diameter 14 mm) and not dilated (left ventricular end-diastolic diameter 47 mm) left ventricle with normal left ventricular ejection fraction and no wall motion abnormalities. There was mild atrioventricular valve regurgitation. However, the main pathological finding using TTE was large circumferential pericardial effusion (23 mm maximum circumference in front of the right ventricle [RV] and posteriorly) leading to impaired filling demonstrating tamponade physiology as indicated by diastolic RV collapse, systolic right atrium (RA) collapse, and dilated inferior vena cava (Figures 2(a)–2(c)). Consequently, immediate pericardiocentesis was performed, which yielded 800 mL of bloody-tinged pericardial drainage. Importantly, following pericardiocentesis, chest pain and dyspnea of the patient stopped and troponin I values declined during the further course of hospitalization. Laboratory analyses of the effusion showed an exudate with elevated protein, leukocytes, cholesterol, glucose, and amylase. Microbiology analyses of the pericardial effusion, however, showed no bacterial growth in solid, liquid, aerobic, and anaerobic cultures. Particularly, we did not detect mycobacteria tuberculosis using Ziehl-Neelsen staining. In addition, interferon-*γ* release assay as well as long-term cultures appeared to be negative for tuberculosis. To exclude possible organ tuberculosis, we performed chest and abdominal computed tomography (CT). Here, we only detected residual circumferential pericardial effusion (Figure 2(d)) and a modest pneumonic infiltrate of the left lower lobe (LLL) of the lung. Consequently, intravenous empiric antibiotic treatment with the ureidopenicillin piperacillin was started and the patient received ibuprofen, a nonsteroidal anti-inflammatory drug, in view of the pericardial effusion.

**Figure 3:**
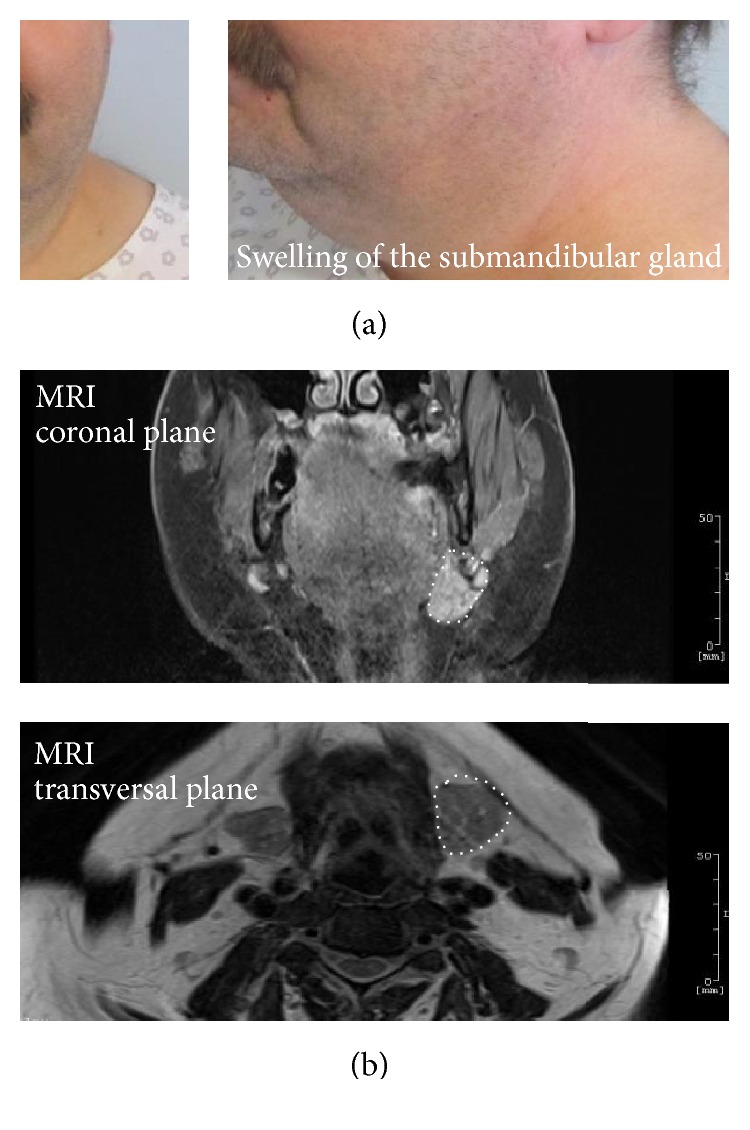
Acute mumps infection involving swelling of the left submandibular gland. (a) Photographs from the left submandibular region of the patient show a superior palpable tumor. (b) MRI reveals an enlarged left submandibular gland with contrast agent accumulation. The left submandibular gland is highlighted by a dotted white line.

To uncover the cause of acute pericardial tamponade in the setting of systemic inflammation, we performed extensive microbiology analyses: antistreptolysin, parvovirus B19,* Borrelia burgdorferi*, coxsackievirus, adenovirus, and influenza A/B immunoglobulin (Ig) titers appeared normal. However, we detected elevated mumps IgM (1.11 AU, <1.00 AU). A later performed control analysis confirmed this result as we detected even increasing IgM titers (1.53 AU, <1.00 AU) indicating an acute mumps virus infection. Notably, the patient did not receive mumps vaccination and did not suffer from mumps before. Importantly, we also measured about 40 times elevated mumps IgG titers (10000 AU, <230 AU), although mumps RNA was not detected in the saliva, blood, or pericardial effusion. To exclude false-positive mumps IgM titers, we performed analyses for measles virus, respiratory syncytial virus (RSV), and parainfluenza type 1, 2, and 3 viruses as these also belong to the family of Paramyxoviridae and can cause false-positive mumps Ig titers [2]. All three analyses appeared negative. Consistent with an acute mumps infection, we also noticed a left submandibular tumor during clinical routine investigations (Figure 3(a)) and magnetic resonance imaging (MRI) of the head-neck-throat region revealed an enlarged and contrast agent-accumulating left submandibular gland (Figure 3(b)) and two augmented lymph nodes near the parotid gland.

Upon antibiotic treatment and supportive care, inflammation markers (CRP, PCT, and leukocytes) decreased. However, complaints about sore throat, headache, fatigue, and sweats persisted for more than 4 days. Control TTE after six weeks revealed only minimal residual effusion without evidence of hemodynamic compromise. At this time point, laboratory analyses were inconspicuous and the patient denied chest pain and dyspnea.

## 3. Discussion

Here, we report coassociation of an acute mumps infection and a life-threatening pericardial tamponade in a 51-year-old man. We performed chest and abdominal CT imaging to exclude organ tuberculosis and pathological traumatic residues that could have led to the present pericardial tamponade, since in 2010 the patient underwent small lung contusion and consecutive pneumomediastinum. However, although the CT was inconspicuous concerning these, we cannot fully rule out the notion that the accident in 2010 predisposed the patient to the development of an acute pericardial tamponade upon mumps infection. Chest CT imaging revealed a small infiltrate of the LLL of the lung. However, we could not detect any bacteria in the sputum, blood, or pericardial effusion. Concerning this, mumps virus was also reported to induce pneumonias that could evoke bacterial superinfections [6]. On the other hand, another possibility could be that the patient indeed first suffered from bacterial pneumonia that led to transient immunosuppression favouring breakthrough of an acute mumps infection.

In our case, mumps RNA could not be detected in the saliva, blood, or pericardial effusion. However, this is not unusual as reverse transcriptase-polymerase chain reaction (RT-PCR) analyses in advanced-stage mumps patients are frequently negative, since the virus RNA can be isolated almost exclusively at symptom onset followed by a rapid decline over the next 10 days indicating a narrow diagnostic window for mumps RNA detection by RT-PCR [2, 5]. Concerning this, our patient already suffered at least two weeks from the infection. In addition to the challenging RNA validation, detection of mumps IgM titers in patients seropositive after prior infection or vaccination is also difficult, since IgM titers do not necessarily increase again with a reinfection [11]. However, detection of very high IgG titers occurs regularly and can be regarded as a reliable marker especially for secondary mumps infections even in the absence of a positive PCR result [12]. In our case, the patient did not receive mumps vaccination, assured us that he never suffered from mumps before, and showed elevated IgM and especially increased IgG titers. As mumps IgM titers can be false positive in patients suffering from other Paramyxoviridae infections, we excluded measles, RSV, and parainfluenza infections [2]. In view of the serological analyses, the acute mumps infection in our patient could be a reinfection after the patient suffered from an asymptomatic disease course years ago.

State-of-the-art clinical case definition of mumps involves an acute onset of unilateral or bilateral swelling of the parotid or other salivary glands without any other apparent cause [1]. Our patient showed no painful and swollen parotid gland(s) as also other publications reported before [1, 2]. However, in line with the current case definition, we detected an enlarged and contrast agent-accumulating left submandibular gland using MRI scans.

In conclusion, our case highlights the importance of considering mumps infection commonly seen as typical childhood disease also in older patients presenting with acute cardiac symptoms. Thereby, our case supports the age shifting hypothesis stating that acute mumps infections are not frequently occurring in children but instead in teenagers and adults today. Suitable, life-threatening complications by the disease are found more often in adults, whereas children in western civilizations have an adequate and sufficient vaccination status [1, 2, 4, 5]. In view of life-threatening disease courses, mumps should always be considered as a differential diagnosis in patients presenting with (peri)myocarditis and/or pericardial effusion and coincident viral infections.
